# *mIDH*-associated DNA hypermethylation in acute myeloid leukemia reflects differentiation blockage rather than inhibition of TET-mediated demethylation

**DOI:** 10.15698/cst2017.10.106

**Published:** 2017-09-20

**Authors:** Laura Wiehle, Günter Raddatz, Stefan Pusch, Julian Gutekunst, Andreas von Deimling, Manuel Rodríguez-Paredes, Frank Lyko

**Affiliations:** 1 Division of Epigenetics, DKFZ-ZMBH Alliance, German Cancer Research Center; 69120 Heidelberg, Germany.; 2 German Consortium of Translational Cancer Research (DKTK), Clinical Cooperation Unit Neuropathology, German Cancer Research Center (DKFZ), Heidelberg, Germany.; 3 Department of Neuropathology, Institute of Pathology, Ruprecht-Karls-University Heidelberg, Heidelberg, Germany.

**Keywords:** mIDH, DNA methylation, AML, TET enzymes, differentiation, D-2-hydroxyglutarate

## Abstract

Isocitrate dehydrogenases 1 and 2 (*IDH1/2*) are recurrently mutated in acute myeloid leukemia (AML), but their mechanistic role in leukemogenesis is poorly understood. The inhibition of TET enzymes by D-2-hydroxyglutarate (D-2-HG), which is produced by mutant *IDH1/2* (*mIDH1/2*), has been suggested to promote epigenetic deregulation during tumorigenesis. In addition, *mIDH* also induces a differentiation block in various cell culture and mouse models. Here we analyze the genomic methylation patterns of AML patients with *mIDH* using Infinium 450K data from a large AML cohort and found that *mIDH* is associated with pronounced DNA hypermethylation at tens of thousands of CpGs. Interestingly, however, myeloid leukemia cells overexpressing *mIDH*, cells that were cultured in the presence of D-2-HG or TET2 mutant AML patients did not show similar methylation changes. In further analyses, we also characterized the methylation landscapes of myeloid progenitor cells and analyzed their relationship to *mIDH*-associated hypermethylation. Our findings identify the differentiation state of myeloid cells, rather than inhibition of TET-mediated DNA demethylation, as a major factor of *mIDH*-associated hypermethylation in AML. Furthermore, our results are also important for understanding the mode of action of currently developed *mIDH* inhibitors.

## INTRODUCTION

Isocitrate dehydrogenase enzymes IDH1 and IDH2 are two closely related enzymes that convert isocitrate to α-ketoglutarate in the tricarboxylic acid (TCA) cycle. The genes encoding IDH1 and IDH2 are recurrently mutated in different cancer entities, such as tumors of the central nervous system, acute myeloid leukemia (AML), cholangiocarcinoma and others 1. Cancer-associated mutations occur at specific amino acids of the active site and confer a neomorphic function to IDH, resulting in the production of D-2-hydroxyglutarate (D-2-HG;^2^). This metabolite has been suggested to competitively inhibit α-ketoglutarate dependent dioxygenases, including protein hydroxylases, histone demethylases and DNA demethylases 3,4. Furthermore, mIDH enzymes lose the ability to produce α-ketoglutarate and NADPH 5, resulting in the disturbance of the TCA cycle and cellular metabolic homeostasis. Consequently, defects of various cellular processes such as differentiation, epigenetic modification, growth factor dependence, DNA damage response, hypoxia signaling, mitochondrial respiration, and apoptosis have been reported in the presence of mIDH proteins 5,6,7,8,9,10,11.

Abnormal DNA methylation patterns have been described as hallmarks of many cancers 12. In human AML and lower-grade glioma, global pathogenic DNA hypermethylation has been associated with the presence of *mIDH *10,13,14,15,16,17,18,19,20. The "CpG island methylator phenotype" (CIMP), a specific hypermethylation signature of CpG islands observed in a subset of cancer entities, was suggested to be directly caused by *mIDH1* expression in glioma 21. Mechanistically, it was hypothesized that TET enzymes, which rely on α-ketoglutarate to initiate DNA demethylation via oxidative conversion of 5-methylcytosine to 5-hydroxymethylcytosine (5hmC), might be inhibited by D-2-HG-producing mIDH and thus be responsible for the observed genomic hypermethylation 15,21. In this context, a critical tumor promoting role was attributed to mIDH-dependent DNA hypermethylation.

Mutated IDH enzymes have also become attractive candidates for oncology drug development and several small molecule inhibitors are currently tested in preclinical and clinical research 17,22,23,24,25,26,27,28,29,30,31. In this context, the reversal of mIDH-associated phenotypes and methylation patterns is considered a key molecular endpoint and biomarker 9,17,32. However, the reported effects of mIDH inhibitors on genomic DNA methylation patterns appeared to be rather moderate 17,22,23,26. These observations suggest that mIDH enzymes might promote tumor growth through mechanisms other than the reported inhibition of TET enzymes 33. Furthermore, these findings emphasize the need to better understand the contribution of mIDH to DNA methylation changes and ultimately tumorigenesis.

DNA methylation is a dynamic epigenetic modification that undergoes widespread changes during mammalian development, cellular lineage commitment and differentiation 34. The hematopoietic system is an excellent model for the analysis of differentiation-associated changes of methylation patterns due to its well characterized differentiation stages and corresponding surface markers allowing the examination of homogeneous primary cell populations 35,36. The methylome analysis of sorted human hematopoietic cells has demonstrated directional changes with a global loss of methylation during myeloid differentiation 37. Hypomethylation during myelopoiesis mainly affects differentiation genes with specific functions in the mature cell types as well as transcription factor binding sites and lineage-specific enhancers 37,38,39. In AML, cells of the stem cell or myeloid progenitor cell pool are malignantly transformed and proliferate aberrantly 40.

Previous analyses of mIDH-dependent DNA methylation changes in AML compared unsorted bone marrow aspirates to healthy donor tissue and partially relied on low CpG coverage assays 13,15. To investigate direct mIDH-, mutant TET- and D-2-HG-dependent DNA methylation changes we analyzed methylation array data of AML patients and HL-60 cells expressing *mIDH*. The presence of mIDH in AML patients was associated with genomic hypermethylation. However, this effect could not be reproduced in *IDH2 *R140Q expressing or D-2-HG treated HL-60 cells or in *TET2* mutant patients. Instead, we found that methylation landscapes of *mIDH* carrying patients resembled normal myeloid progenitor cell methylomes. This suggests that altered differentiation states rather than inhibition of DNA demethylation define the mIDH-dependent methylation landscape.

## RESULTS

### Genomic hypermethylation in AML patients with *mIDH*

To characterize the methylation patterns associated with neomorphic *IDH* mutations, we analyzed a published dataset of adult *de novo* AML 20. These patients had been clinically annotated and their bone marrow aspirates had been subjected to whole-genome or whole-exome sequencing, RNA sequencing and DNA methylation analysis using Illumina Infinium HumanMethylation450 arrays. We extracted methylation profiles of 28 patients with mutations in either *IDH1* (R132) or *IDH2* (R140/R172) - herein referred to as *mIDH* - and 112 patients with *IDH* wildtype (WT) status. The raw intensity data files were normalized, quality-filtered and statistically analyzed using a standard analytical pipeline 41.

**Figure 1:**
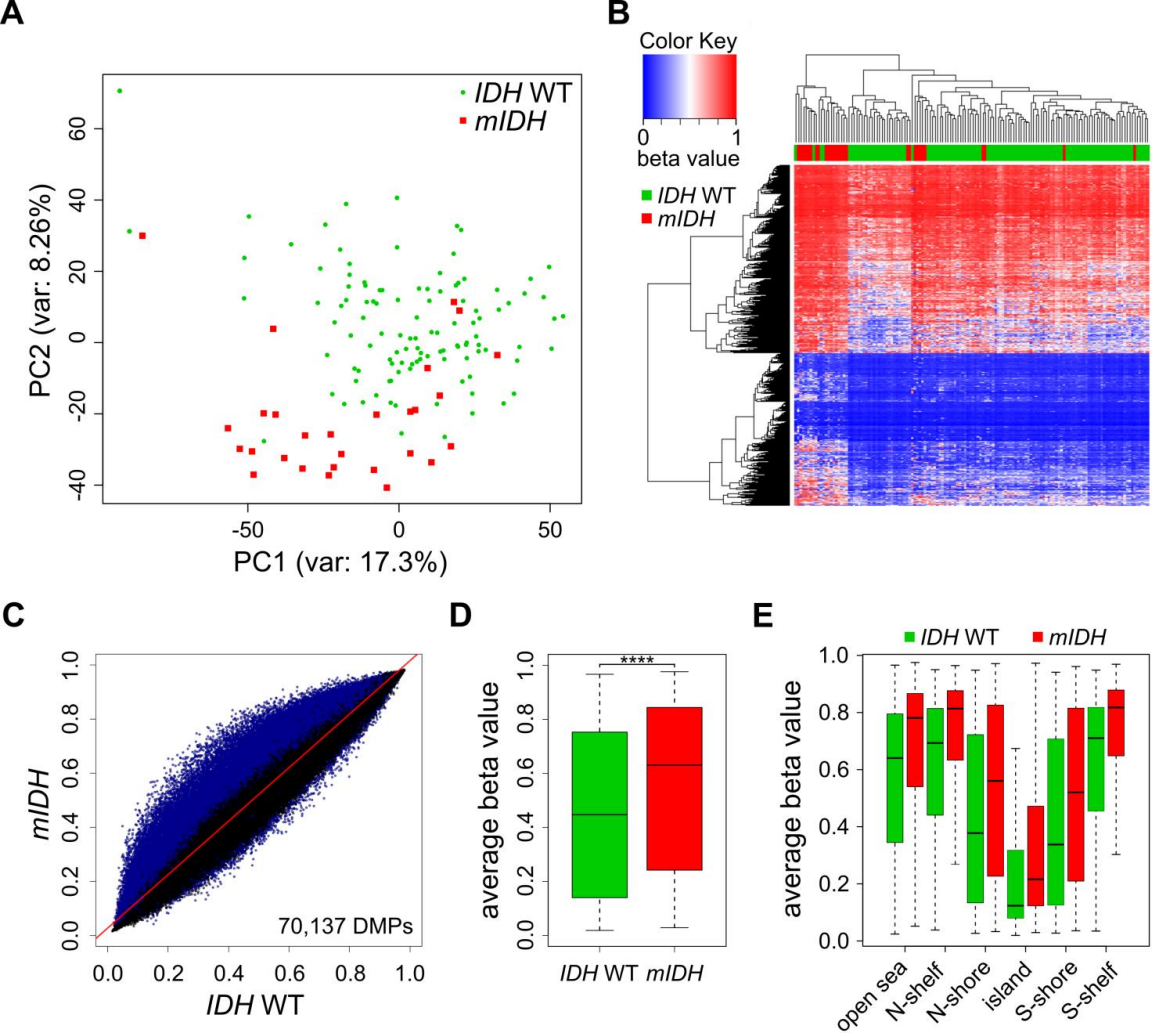
FIGURE 1: AML patients with *mIDH* display genomic hypermethylation. **(A)** Principal Component Analysis of methylomes of 28 AML patients with *mIDH* and 112 AML patients with *IDH* WT status using all 450K probes retained after quality filtering. **(B)** Heatmap of the 5000 most significantly (adj. *P*<0.05) differentially methylated 450K probes between the two patient groups. Each column represents one patient and each row one probe. Dendrograms of patients and probes were obtained using hierarchical clustering by similarity. The color scale indicates beta values. **(C)** Scatterplot of the beta values of all 450K probes comparing *mIDH* to *IDH* WT patients. Each probe retained after quality filtering is represented by a dot, with significantly (adj. *P*<0.05) differentially methylated probes (DMPs) depicted in blue. **(D)** Boxplot showing the average beta values of all the significantly differentially methylated probes in the two groups. The difference between the two groups was highly significant (**** *P*<0.0001). **(E)** Boxplot showing the average beta values of the significantly differentially methylated probes associated with different epigenomic features in *mIDH* and *IDH* WT patients. All differences observed between the two patient groups were highly significant (*P*<0.0001).

Comparison of the two patient groups by unsupervised Principal Component Analysis (PCA) using all 450K probes separated *mIDH* from *IDH* WT patients with few exceptions (**Fig. 1A**), suggesting the presence of specific changes in *mIDH*-associated methylation profiles. We also performed hierarchical clustering based on the 5000 most significantly differentially methylated (adj. *P*<0.05) probes. This again correctly segregated the majority of the AML patients according to *IDH* mutational status, with prominent hypermethylation in the cluster defined by *mIDH* patients (**Fig. 1B**). To assess the methylation changes in *mIDH* patients in more detail, we plotted the beta values of all 70,137 significantly differentially methylated (adj. *P*<0.05) probes in the two patient groups. The vast majority (68,863) of these probes appeared hypermethylated in patients with *mIDH *(**Fig. 1C**). Most differentially methylated probes had an intermediate methylation level in *IDH* WT AML patients which was increased by approximately 20% in *mIDH *patients and this increase was highly significant (**Fig. 1D**; *P*<0.0001). Our analysis of epigenomic features in *mIDH* and *IDH* WT patients revealed that CpG islands, shores, shelves and open sea were all significantly hypermethylated in *mIDH* patients (**Fig. 1E**).

We also analyzed the possibility that mutations in *DNMT3A* might influence our PCA and clustering analysis. We therefore compared 32 *DNMT3A* mutated patients (12 had co-occurring *mIDH*) to 108 patients with wildtype *DNMT3A *(20 had co-occurring *mIDH*) from this cohort. The general effect of *DNMT3A *mutation was genomic hypo-methylation (23,795 hypomethylated out of 26,334 differentially methylated probes), leading to a modest, but significant reduction of average beta values (Figure S1A, B).

Interestingly, many of the outliers in our original PCA (**Fig. 1A**) had co-occurring *DNMT3A* mutations (Figure S1C) and when *mDNMT3A* patients were removed from the initial patient set for hierarchical clustering, segregation according to *IDH* mutation status was further improved (Figure S1D). Taken together, these results confirm widespread CpG hypermethylation associated with neomorphic *IDH1/2 *mutations in AML.

### Methylation analysis of a cellular model expressing *mIDH2*

**Figure 2:**
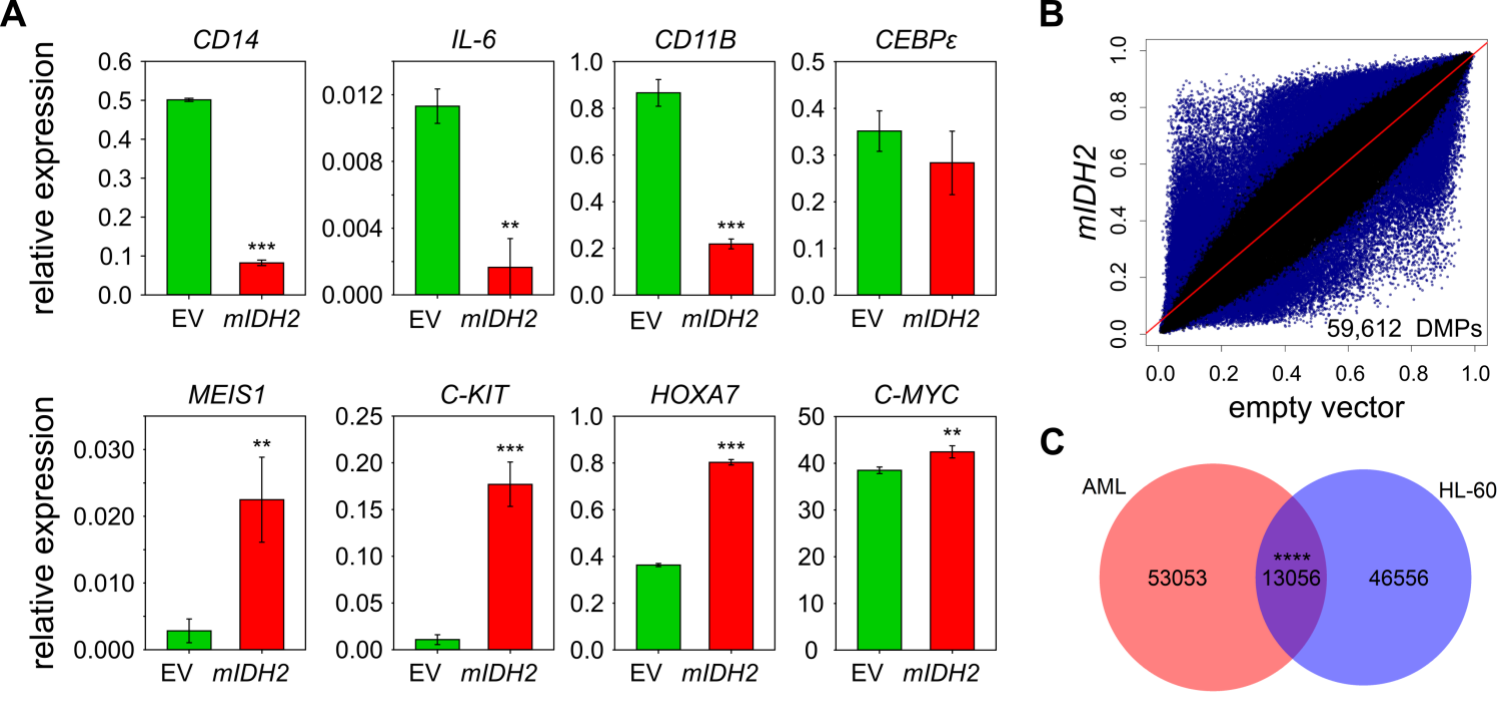
FIGURE 2: Genomic methylation profiles of *mIDH* AML patients and HL-60 cells expressing *IDH2* R140Q are distinct. **(A)** Expression analysis of myeloid progenitor and differentiation genes by qRT-PCR in HL-60 cells transduced with empty vector (EV) or *mIDH2. *Expression is shown relative to* ACTB *transcript levels. Error bars indicate standard deviation (n=3). Statistical significance was calculated using a two-sided Student's *t*-test (*** *P*<0.001, ** *P*<0.01). **(B)** Scatterplots comparing average beta values for all probes common to 450K and EPIC chip between HL-60 cells transduced with *mIDH2* or empty vector. Each dot represents one probe with DMPs colored in blue. Two biological replicates were analyzed independently on the 450K chip per condition. **(C)** Venn diagram showing the overlap between DMPs found upon presence of *mIDH* in the AML patient dataset and the HL-60 cells. The overlap between the two groups was minor, but greater than expected by chance using the hypergeometric test (**** *P*<0.0001).

In parallel experiments, we generated HL-60 leukemia cells that had either *mIDH2* (R140Q) or the corresponding empty vector stably integrated into their genomes. Introduction of *mIDH2* resulted in a fivefold increase of *IDH2 *mRNA expression compared to empty vector (Figure S2A). Consistently, D-2-HG levels in the cell culture medium of *mIDH2* expressing cells were elevated more than 30-fold to approximately 11 μM relative to cells transduced with empty vector (Figure S2B). Interestingly, this did not lead to a reduction of TET-dependent global 5hmC levels (Fig. S2C). Furthermore, *mIDH2* expressing HL-60 cells showed no overt morphological changes (Figure S3A) and cumulative population doubling measurements indicated that *mIDH2* expressing cells had an unchanged proliferation rate (Figure S3B). However, expression analysis of hematopoietic stem cell and myeloid differentiation genes indicated several notable changes: Markers of differentiated myeloid cell states such as *CD11B* were downregulated, while genes expressed in myeloid progenitor cells such as *C-KIT* were upregulated in *mIDH2* expressing cells compared to the empty vector (**Fig. 2A**), indicating that *mIDH2* expressing HL-60 cells display gene expression changes reminiscent of a less differentiated state. Infinium methylation analysis of two biological replicates per condition showed diverse methylation changes with both hyper- and hypomethylation in HL-60 cells expressing *mIDH2 *(**Fig. 2B**), that were clearly different from the specific hypermethylation observed in *mIDH* AML patients (compare to **Fig. 1C**).

Partially methylated domains are known to acquire hypomethylation in cultured cells 42 and coincide with lamina-associated domains (LADs; 43). To exclude that these regions are responsible for the observed hypomethylation in *mIDH2* cells, we removed LAD-associated probes from our analysis. This did not affect the overall result (Fig. S4), indicating that hypomethylation upon expression of *mIDH2* occurs outside of LADs. We also identified the probes that were commonly differentially methylated in *mIDH* AML patients and *mIDH2* HL-60 cells, which failed to identify a major overlap (**Fig. 2C**). Together, these results suggest that the specific DNA hypermethylation pattern observed in *mIDH* patients cannot be faithfully recapitulated by *mIDH2* overexpression in a cell-based model.

### *TET2 *mutations in AML are not associated with global DNA hypermethylation

**Figure 3:**
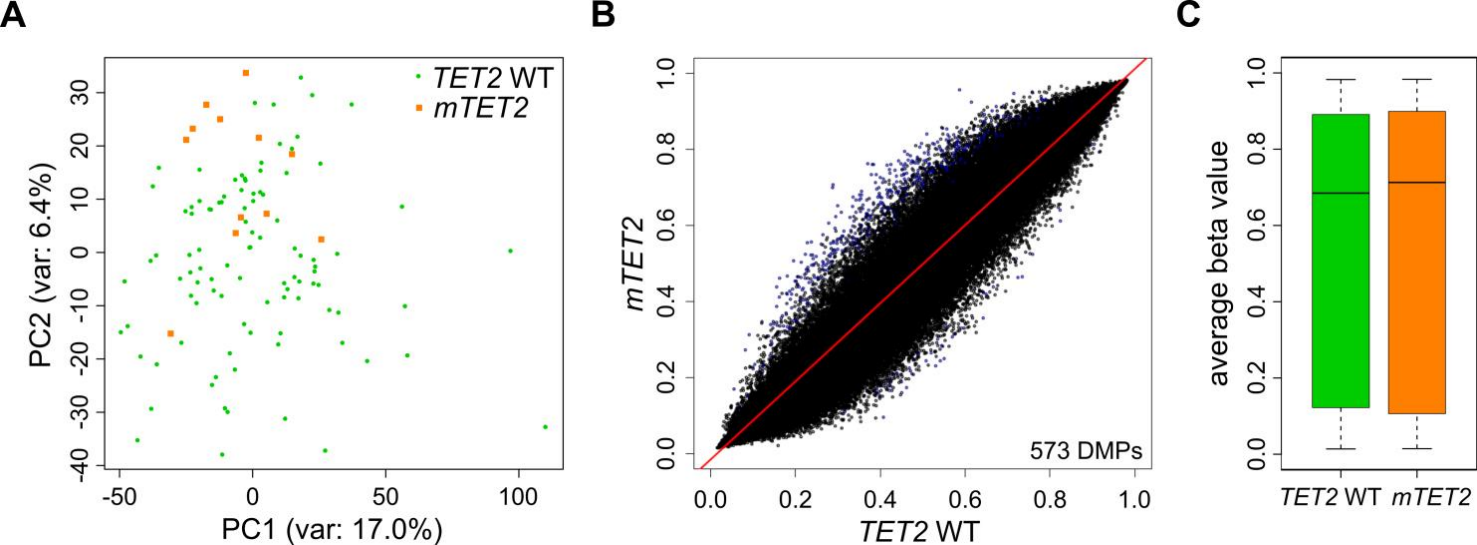
FIGURE 3: *mIDH*-associated hypermethylation is not replicated by *TET2* mutation in AML patients. **(A)** Principal Component Analysis of *TET2 *WT and *TET2* mutant AML patients using all probes retained after quality filtering. No clear separation of the two patient groups was achieved. **(B)** Comparison of AML patients with and without *TET2* mutations by scatterplot. The average beta value of each 450K probe in the two groups is represented as a single dot. DMPs are colored in blue. *IDH* mutant patients were removed from the analysis. **(C)** Boxplot of average beta values of all probes in *TET2 *WT and *TET2* mutant AML patients (*P*=0.52).

It has been suggested that TET-mediated DNA demethylation is inhibited by mutations in *IDH* genes, but the observed (hydroxy)methylation changes were determined by indirect assays and appeared rather moderate 15,21. In our hands, overexpression of *mIDH2* in HL-60 cells also did not influence 5hmC abundance in genomic DNA (Fig. S2C), suggesting an oxidation-independent mechanism of *mIDH*-associated hypermethylation. Additional studies have found contradictory results with regard to methylation changes in *TET2* mutated myeloid malignancies 44,45,46. To analyze potential TET-dependent hypermethylation effects, we extracted patients with mutations in the *TET2 *gene from the TCGA cohort. These 12 patients presented with nonsense or frameshift mutations occurring in front of the C-terminal catalytic domain, thus destroying the enzymatic function of TET2. The comparison of methylation patterns from the 12 *TET2* mutant patients to 100 *TET2* and *IDH1/2* WT patients did not separate *TET2* WT and *TET2* mutant patients into distinct clusters in a PCA with all probes (**Fig. 3A**). Also, we identified only a small fraction of differentially methylated probes (**Fig. 3B**) which resulted in a very minor and statistically not significant (*P*=0.52) increase in the average methylation ratio of *TET2* mutant patients (**Fig. 3C**). Together, these data suggest that there are no systematic large-scale DNA methylation changes associated with *TET2* mutations in AML, which contrasts the observed *mIDH*-associated differences. Consequently, it appears unlikely that inhibition of TET2 is a major factor contributing to *mIDH*-associated DNA hypermethylation.

**Figure 4:**
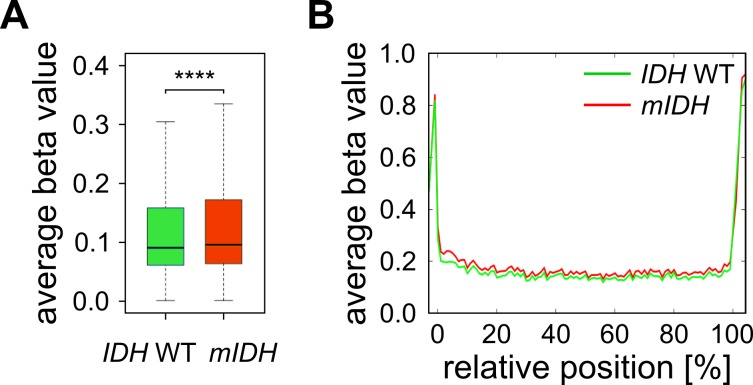
FIGURE 4: TET-dependent DNA methylation canyons are not specifically affected by *mIDH*-associated hypermethylation in AML. **(A)** Average methylation ratios of canyon-associated probes in *mIDH* and *IDH* WT patients. The difference between the two groups was highly significant (**** *P*<0.0001). **(B)** Superposition of all size-normalized canyons depicting average methylation levels of these features in the two patient groups.

Furthermore, we have previously shown that genetic deficiency for *Tet1* and *Tet2* in mouse embryonic fibroblasts leads to specific hypermethylation of DNA methylation canyons 47. Canyons are conserved regions of at least 3.5 kb that are largely depleted of DNA methylation and frequently harbor genes of developmental regulators. They are considerably larger than CpG islands and also contain CpG island shore and shelf regions 48. Assuming that 2-HG inhibits TET enzymes, we expected canyons to be specifically targeted by hypermethylation in *mIDH* expressing AML patients. We used a high-coverage whole genome bisulfite sequencing dataset from an *IDH* WT AML patient 49 to identify 1711 canyons and 26,117 associated Infinium 450K probes. Average methylation ratios of these canyon-associated probes were only minimally (delta beta 0.014), but significantly increased in patients with *mIDH* (**Fig. 4A**). The small gain in methylation was evenly distributed over the length of the canyon and not enriched at canyon borders (**Fig. 4B**), which is distinct from *Tet1/2*-deficient cells 47. These findings again support the notion that *mIDH*-associated genomic hypermethylation is mediated by factors other than TET inhibition.

### D-2-hydroxyglutarate does not induce genomic hypermethylation in cancer cells

**Figure 5:**
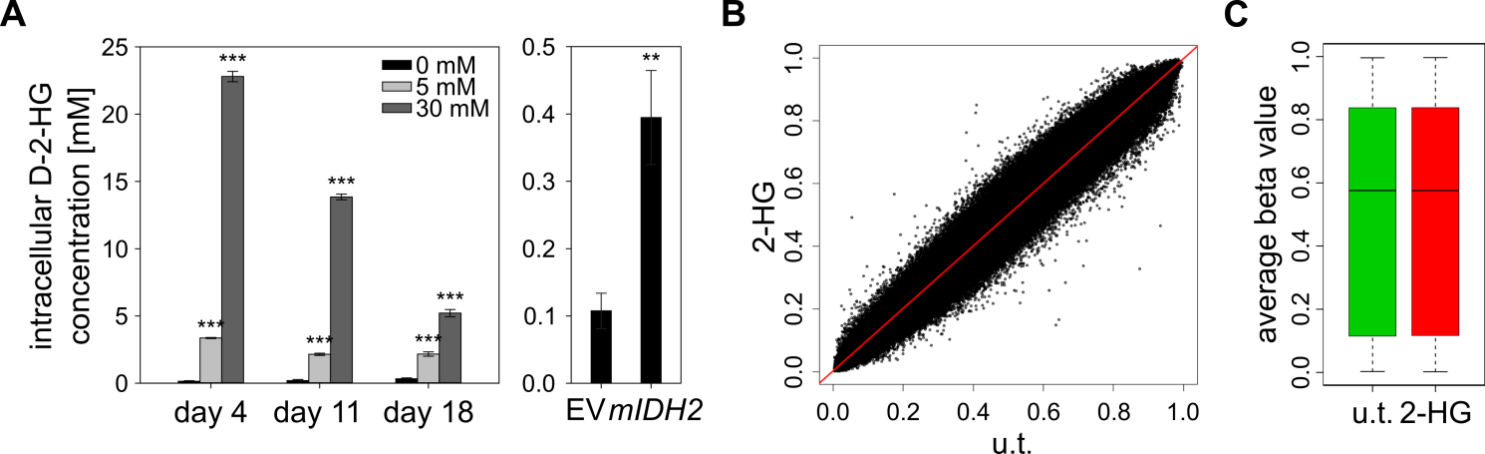
FIGURE 5: D-2-HG does not induce genomic hypermethylation in cultured cells. **(A)** Measurement of intracellular D-2-HG concentrations using an enzymatic conversion assay 50. HL-60 cells were cultured in medium containing 5 or 30 mM synthetic D-2-HG and quantifications of intracellular concentrations were performed at the indicated time points. The right panel shows intracellular concentrations in HL-60 cells transduced with empty vector (EV) or *mIDH2*. Statistical significance was calculated using the two-sided Student's *t*-test (*** *P*<0.001 and ** *P*<0.01). **(B)** Comparison of methylomes from D-2-HG treated (2-HG; 30 mM for 21 days) and untreated (u.t.) HL-60 cells by scatterplot. No significantly changed probes (adj. *P*<0.05) were found analyzing two biological replicates of each condition by EPIC methylation array. **(C)** Boxplot of average beta values of all probes in untreated and D-2-HG treated (30 mM for 21 days) HL-60 cells (*P*=0.17).

D-2-HG produced by mIDH enzymes has been shown to inhibit the hydroxylation activity of α-ketoglutarate dependent dioxygenases, including the TET enzymes 3,4. However, these studies relied on 5hmC immunostainings upon TET overexpression or on *in vitro* enzymatic assays achieving only partial inhibition of TET activity, indicating that 2-HG is only a weak inhibitor of TET enzymes. Furthermore, the ability of D-2-HG to inhibit DNA demethylation in human cells has not been established yet. To mimic the conditions observed in *mIDH* AML patients, we incubated HL-60 cells in medium supplemented with a high concentration (30 mM) of synthetic D-2-HG. These cells had a significantly reduced proliferation rate (Fig. S5) but remained viable. Measurement of intracellular D-2-HG demonstrated that HL-60 cells imported the metabolite, resulting in intracellular concentrations that strongly exceeded those obtained by overexpression of *mIDH2* (**Fig. 5A**). We next analyzed genomic DNA from cells treated with 30 mM of D-2-HG for three weeks in two independent experiments using Infinium methylation arrays. Comparison of D-2-HG treated with untreated HL-60 cells identified no significantly DMPs (**Fig. 5B**). Furthermore, the average methylation level was unchanged in treated compared to untreated cells (**Fig. 5C**; *P*=0.17). Thus, our data strongly suggest that D-2-HG is not sufficient to induce DNA hypermethylation in HL-60 cells. In addition, these findings confirm the notion that *mIDH*-associated hypermethylation is not caused by ineffective TET-mediated DNA demethylation.

### *mIDH*-associated methylation patterns resemble myeloid progenitor methylomes

**Figure 6:**
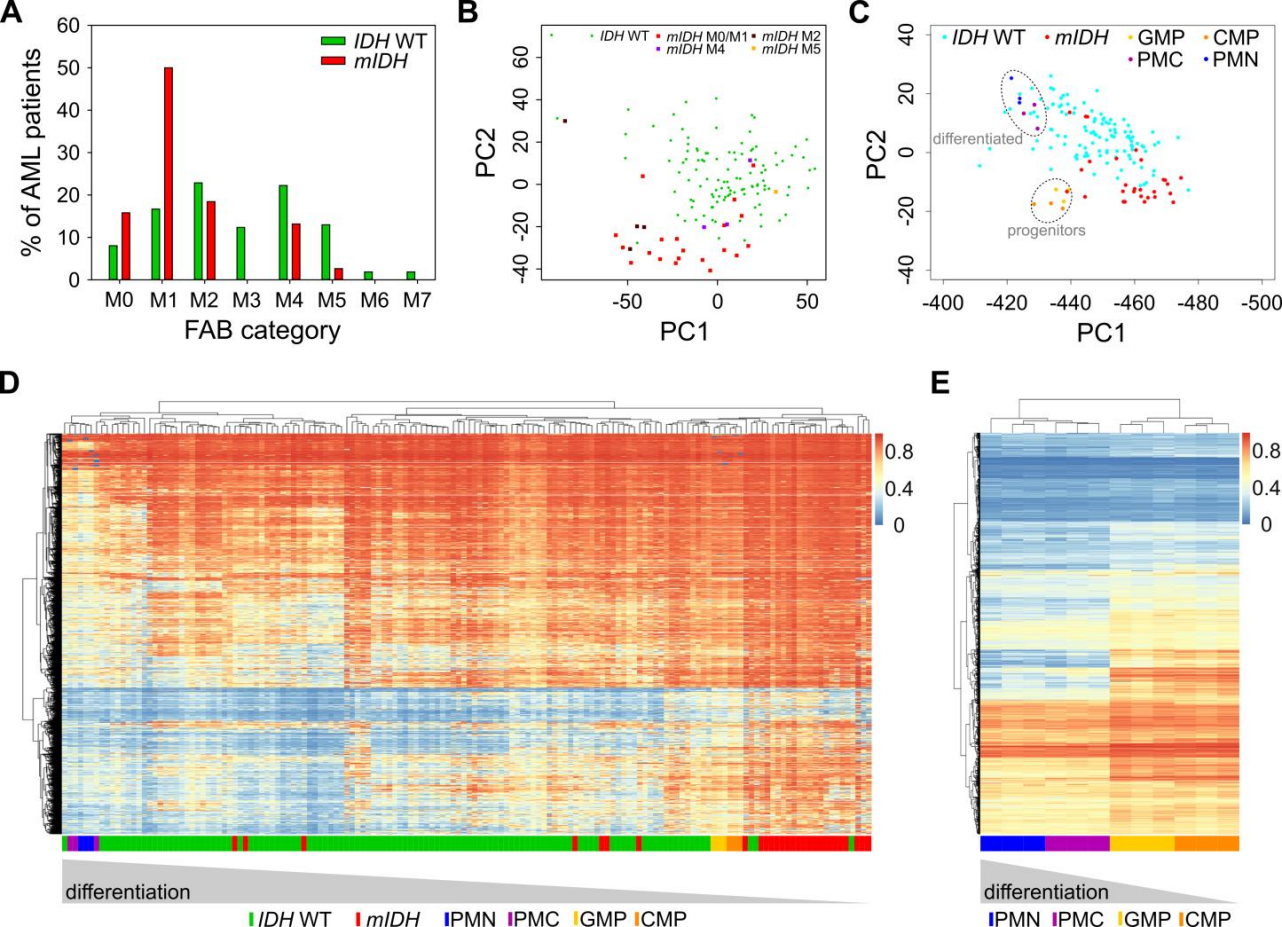
FIGURE 6: *mIDH*-associated hypermethylation reflects enriched hypermethylated hematopoietic progenitor populations rather than a pathological hypermethylator phenotype. **(A)** Analysis of the distribution of the French-American-British (FAB) categories assigned in the AML dataset from TCGA within the *IDH* WT and *mIDH* patient groups. **(B)** PCA as in Fig. 1A. Different FAB categories in the *mIDH *patient group are colored as indicated. Variances captured by PC1 and PC2 were 17.3% and 8.3%, respectively. **(C)** PCA with 450K profiles from sorted human cells of four hematopoietic differentiation stages 38. TCGA AML patient profiles of *mIDH*/*IDH* WT patients were projected on top of the clusters generated by analysis of the four cell types. CMP: common myeloid progenitor, GMP: granulocyte macrophage progenitor, PMC: promyelocyte, PMN: polymorphonuclear/terminally differentiated bone marrow neutrophil. **(D)** Heatmap of the 10,000 most differentially methylated probes between the CMP/GMP and PMC/PMN populations in the AML cohort of *mIDH*/*IDH* WT patients. Each row shows one probe and each column one patient. A hierarchical cluster dendrogram showing similarities between samples is depicted on the top. The color scale indicates beta values. **(E)** Heatmap and hierarchical cluster dendrogram of human hematopoietic cell types using the 10,000 most differentially methylated probes between *mIDH* and *IDH* WT AML patients. Each row shows one probe and each column one sample. The color scale indicates beta values.

Methylome analysis of sorted hematopoietic cell types from various differentiation stages demonstrated that myeloid differentiation is associated with a global loss of DNA methylation 37,39. AML samples are usually unpurified bone marrow aspirates with a high blast count comprising all kinds of hematopoietic differentiation stages. Since previous reports have shown that mIDH blocks differentiation 9,15,51,52,53, we reasoned that *mIDH*-associated genomic hypermethylation might simply reflect physiological methylation patterns of less differentiated myeloid progenitor cell types. We therefore analyzed the distribution of French-American-British (FAB) classes, which are routinely used to morphologically distinguish the predominant cell type and maturity of AML. This showed a clear enrichment of the relatively undifferentiated M1 subclass within the *mIDH* patients compared to the *IDH* WT cohort (**Fig. 6A**). Undifferentiated acute myeloblastic leukemia (M0) was also more frequent in the *mIDH* group than in the *IDH* WT group, while the remaining classes were relatively rare in *mIDH* patients or not present at all (**Fig. 6A**). Moreover, PCA showed that patients with a higher grade of maturation (M2 - M5) clustered together with the *IDH* WT patients (**Fig. 6B**), indicating that AML cells which manage to mature in the presence of *IDH* mutations adopt methylation profiles similar to *IDH* WT AML methylomes.

We also examined methylation patterns of four purified cell types of the human hematopoietic lineage (CMP, GMP, PMC and PMN), which were analyzed previously by 450K methylation arrays 38 and could therefore be directly integrated into our analysis. PCA segregated the less differentiated CD34 positive (CMP and GMP) from the more differentiated CD34 negative (PMC and PMN) samples. When AML methylation profiles were projected on top of these reference methylation profiles, *mIDH* samples clustered together with the undifferentiated cells, whereas *IDH* WT samples appeared closer to the differentiated cell types (**Fig. 6C**). Finally, we also subjected the 10,000 most differentially methylated probes between the progenitor cells (GMPs and CMPs) and the differentiated cells (PMCs and PMNs) to hierarchical clustering. This separated the two differentiation stages from each other and placed most *mIDH* AML patients in the same branch as the less differentiated myeloid progenitor cells (**Fig. 6D**). When we used the 10,000 most differentially methylated probes between *mIDH* and *IDH* WT patients to cluster the sorted hematopoietic cell types, CD34 positive progenitors were again separated from CD34 negative cells. Also the GMP and CMP samples were placed in different branches consistent with their differentiation state (**Fig. 6E**). Together, these findings identify physiological methylation states of arrested myeloid progenitor cells as a major source of *mIDH*-associated hypermethylation in AML.

## DISCUSSION

DNA hypermethylation is a key feature of many cancer epigenomes but its origin and functional contribution to tumorigenesis remain controversial. Here, we have analyzed a large AML cohort from TCGA and found genomic hypermethylation associated with *IDH1/2* mutations. This is consistent with previous studies and findings in other tumor entities such as lower-grade glioma or cholangiocarcinoma 15,21,54. However, AML-specific hypermethylation patterns could not be identified in cells with *mIDH2* overexpression or D-2-HG treatment. In conjunction with the paucity of large-scale DNA methylation changes in *TET2* mutant AML patients, this critically challenges the current paradigm of mIDH-dependent DNA hypermethylation by D-2-HG-mediated TET inhibition.

The claim that mutant *IDH* induces hypermethylation through its action on TET enzymes 15,21, would suggest similar phenotypes upon expression of mIDH and loss of TET activity. However, in support of our study there are clinical differences in *TET2* and *IDH* mutant diseases and distinct hematopoietic phenotypes in *Tet2*-deficient and *mIdh* expressing mice. *Tet2*-deficient mice show a more pronounced expansion of the stem cell pool than *mIdh1* expressing animals and additionally display augmented repopulation activity and skewed differentiation 7,10,55,56,57. Moreover, the effect of *TET2* mutations on DNA methylation in human myeloid malignancies remains unclear with some studies identifying hypermethylation 15,45,58,59 and others hypomethylation 44,46. Also, global 5mC levels were not affected in human erythropoietic cell types upon *TET2/TET3* knockdown 60. This is in agreement with our findings and suggests that TET deficiency and *IDH* mutations have distinct epigenetic effects in myeloid malignancies.

While the reported paracrine activity of 2-HG 33 should allow inhibition of TET activity without mIDH, DNA hypermethylation was not detectable in our cellular model. This indicates that hypermethylation may be induced by pathways that are independent of the catalytic activity of mIDH. It should be noted that AML cells with *mIDH* may be exposed to high 2-HG concentrations over a much longer period than several weeks and that they may also be influenced by 2-HG-mediated changes of the stromal niche 61. However, if TET-mediated DNA demethylation was inhibited over several cell divisions, our *in vitro *model should display DNA hypermethylation. Further clarification may require longer 2-HG exposure or appropriate mouse models. Indeed, when 2-HG was administered to different leukemia mouse models over 4 weeks, leukemia-promoting effects were observed, but the metabolite was not sufficient to induce leukemia or change DNA methylation patterns to mimick *IDH1* mutants 33. Also, several reports have shown that the inhibition of D-2-HG production by mIDH inhibitors did not revert global DNA hypermethylation in glioma and AML cells 22,23,26. These observations emphasize that depletion of 2-HG is not sufficient to restore normal methylomes.

An mIDH-dependent differentiation block has repeatedly been reported in different *in vitro* and *in vivo* systems 9,10,15,51,52,53. Accordingly, we found an enrichment of FAB categories representing early differentiation stages in mIDH AML patients. Furthermore, our comparison with sorted myeloid cell types indicated that *mIDH*-associated methylation patterns strongly resemble myeloid progenitor cell types. We therefore propose that *mIDH* expression keeps AML cells in a less differentiated state that is reflected in their methylation landscape. *IDH* mutations are early events in the transformation of blood cells as suggested by analyses of premalignant cells and hematopoietic disorders 1,62,63,64. *mIDH* may thus inhibit differentiation of the mutated progenitor and all clonally derived cells, whereas cells with a wildtype *IDH* allele may retain the capacity to differentiate further. Together with the finding that the TET inhibitor L-2-HG did not recapitulate the differentiation block observed with D-2-HG in cell-based experiments 9, our results suggest a TET-independent mechanism leading to differentiation arrest 3,51,65.

CpG island hypermethylation or more specifically CIMP has been described in many cancers, however it cannot always be correlated with *IDH* or other mutations 66,67,68,69. Furthermore, methylation patterns of B-cell chronic lymphocytic leukemia were described to be highly related to normal methylomes of various differentiation stages 70,71,72. In addition, CIMP was recently described as a cancer-independent feature of proliferating myeloid cells 73, which further challenged the cancer-specificity of this phenotype. Our findings suggest that mIDH-associated hypermethylation in AML reflects the relatively undifferentiated state of the cancer cells, rather than impaired DNA demethylation. Our study thereby furthers the understanding of *mIDH*-associated alterations in DNA methylation and emphasizes the need for a detailed characterization of the 2-HG-dependent and independent functions of mutant IDH enzymes.

## MATERIALS AND METHODS

### Patient data

Raw .IDAT Illumina Infinium 450K methylation array files, clinical patient parameters including FAB categories and mutational status were downloaded from the TCGA database or GDC Legacy Archive 20.

### Cell culture, generation of stable *mIDH2* expressing HL-60 cells and proliferation analysis

HL-60 cells were cultured in RPMI supplemented with 10% (v/v) fetal bovine serum, 100 U/ml penicillin and 100 μg/ml streptomycin. For generation of stable cell lines IDH2 R140Q was cloned into pLVX-IRES-ZsGreen1. HEK293T cells were grown to 90% confluency in 6-well plates and transfected with 2.5 μg of DNA mix consisting of pLVX-IRES-ZsGreen1 (empty vector)/pLVX-IRES-ZsGreen1-mIDH2, psPAX2 and pMD2.G in a ratio of 5:4:1 using lipofectamine 2000 according to the manufacturer´s instructions. After two days viral particles were harvested, filtered (0.45 μm) and 200,000 HL-60 cells/well were resuspended in virus-containing cell culture supernatant for infection. After a few days the top 30% of fluorescent cells were FACS sorted by ZsGreen1 expression to pools of at least 1000 cells. The sorting was repeated several times. For proliferation analysis, at each passage 500,000 cells were seeded in triplicates and after 3 - 4 days cell numbers/well were determined and cells were passaged. The population doubling level was computed from averages of triplicates as described before 74 and the cumulative population doubling level at each passage was plotted against time.

### Cellular 2-HG treatment and quantification

D-2-HG (Sigma) was freshly dissolved in RPMI, the solution was rotated for 30 min at room temperature and sterile-filtrated (0.2 μm). 600,000 cells/well were seeded into a 24-well plate in 1 ml of D-2-HG supplemented medium. Medium was exchanged and cells were reduced to 600,000/well twice per week. Remaining cells were quickly washed in PBS for two times, pelleted and stored at -20°C. Intra- and extracellular D-2-HG quantifications were performed as described previously 50.

### DNA and RNA isolation

Genomic DNA was isolated by lysing freshly prepared cell pellets in pre-lysis buffer (10 mM Tris-HCl (pH 8), 5 mM EDTA, 100 mM NaCl, 1.1% (v/v) SDS, 0.1 mg/ml Proteinase K and 0.04 mg/ml RNAse A) at 37°C. The next day proteins were precipitated by addition of 5 M NaCl and the DNA was isolated by isopropanol precipitation. Total RNA was isolated using TRIzol (Invitrogen).

### cDNA synthesis and gene expression analysis

cDNA was synthesized from 1 μg RNA (QuantiTect Reverse Transcription Kit; Qiagen) following the manufacturer's instructions. Quantitative RT-PCRs were performed in triplicate using Mesa green qPCR mastermix PLUS (Eurogentec) and the Lightcycler 480 Real-Time PCR system (Roche). Expression values were calculated from raw Ct values using the ΔΔCt method and *ACTB* as a reference gene.

### Dotblots and Infinium EPIC methylation assay

Genomic DNA was isolated and 4 μg were used for dotblots as described before 47. For methylation profiling 1 μg of DNA was used. The Infinium MethylationEPIC BeadChip assay was performed by the microarray unit of the DKFZ Genomics and Proteomics Core Facility. Briefly, per experiment and condition two biological replicates were used. Matched untreated or empty vector transduced cells served as controls. 500 ng of high quality genomic DNA were bisulfite converted using the EZ-96 DNA Methylation Kit (Zymo Research) according to the manufacturer's instructions, whole genome amplified, enzymatically fragmented and denatured following the recommendations of the Infinium HD Assay Methylation Protocol Guide (Illumina), and hybridized to the BeadChip.

### Infinium analysis pipeline

The raw intensity data files were normalized, quality-filtered and statistically analyzed as described before 75. In brief, the minfi package 41 was used to load IDAT files into R. Data were normalized with the SWAN method 76 without prior background correction. Cross-reactive and SNP containing probes 77, probes on the sex chromosomes and probes with a low detection P-value (>0.01) were omitted. In order to allow comparisons between 450K and EPIC datasets only probes present on both chips were included. PCA graphs were generated by the plotPCA function provided by the R affycoretools package 78 with all probes used to compute principal components. Heatmaps were created by the heatmap.2 function of the R gplots package 79. For clustering the euclidean distance function was used to compute the distance matrix and the complete linkage method to obtain hierarchical clustering. The RColorBrewer package was used to create the assigned colors 80.

### Mapping of canyons and canyon analysis using 450K data

Canyons were computed as described before 47 using a whole genome bisulfite sequencing dataset from a female AML patient with wildtype IDH genes available from the Blueprint Epigenome Consortium (http://www.blueprint-epigenome.eu; 49). This led to a set of 1,711 canyons covering a total of 26,117 Infinium 450K probes. For further analysis the canyons were size-normalized and the average methylation profiles of IDH WT and mIDH samples were computed.

### Integrated Principal Component Analysis of FANTOM and TCGA data

A PCA of the 450K methylation data of 12 differentiation related blood cell datasets generated as a part of the FANTOM5 project 38 was computed using the R package FactoMineR (http://factominer.free.fr) . TCGA AML patient methylation profiles of mIDH/IDH WT patients were projected onto this PCA using the new basis vectors computed by the PCA.

### Heatmaps and cluster dendrograms

Heatmaps and cluster dendrograms were computed using the R package pheatmap.

### Statistical methods

Differentially methylated probes between wildtype and mutant patients or HL-60 cells were identified by a similar approach as implemented in minfi 41. Briefly, differentially methylated CpGs were detected by a linear model fit of methylation values, which enabled an empirical Bayes moderation of standard errors from estimated methylation differences. Subsequently, P-values were adjusted for false discovery rate using the Benjamini-Hochberg procedure. Probes with an adjusted P-value (adj. *P*) <0.05 were considered as differentially methylated. Significance of alterations in global methylation was inferred by a two-sided, unpaired, Welch-corrected *t*-test. Gene expression and D-2-HG differences were tested using an unpaired, two-sided Student's t-test or Welch Two Sample t-test if variances were unequal. A hypergeometric test was used to calculate the significance level of overlapping probes.

### Data access

Infinium raw data (.IDAT files) of HL-60 cells have been deposited in the ArrayExpress database at EMBL-EBI (http://www.ebi.ac.uk/arrayexpress) under accession number E-MTAB-6059.

## SUPPLEMENTAL MATERIAL

Click here for supplemental data file.

All supplemental data for this article are also available online at http://www.cell-stress.com/researcharticles/midh-associated-dna-hypermethylation-in-acute-myeloid-leukemia-reflects-differentiation-blockage-rather-than-inhibition-of-tet-mediated-demethylation/.

## Abbreviations

2-HG: 2-hydroxyglutarate
5hmC: 5-hydroxymethylcytosine
AML: acute myeloid leukemia
CIMP: CpG island methylator phenotype
DMP: differentially methylated probe
FAB: French-American-British
IDH: isocitrate dehydrogenase
LAD: lamina-associated domain
mIDH: mutant IDH
PCA: Principal Component Analysis
