# Supplementary material for: mIDH-associated DNA hypermethylation in acute myeloid leukemia reflects differentiation blockage rather than inhibition of TET-mediated demethylation
Source: Cell Stress. 2017 Sep 20;1(1):55–67. doi: 10.15698/cst2017.10.106 (PMC6551656; doi:10.15698/cst2017.10.106)
Supplement: Supplementary file 1 [file ces-01-055-s01.pdf]

SUPPLEMENTAL MATERIAL

mIDH-associated DNA hypermethylation in acute myeloid leukemia reflects differentiation blockage rather than inhibition of TET-mediated demethylation

Wiehle et al.

Contents:

Supplemental Figures S1-S5

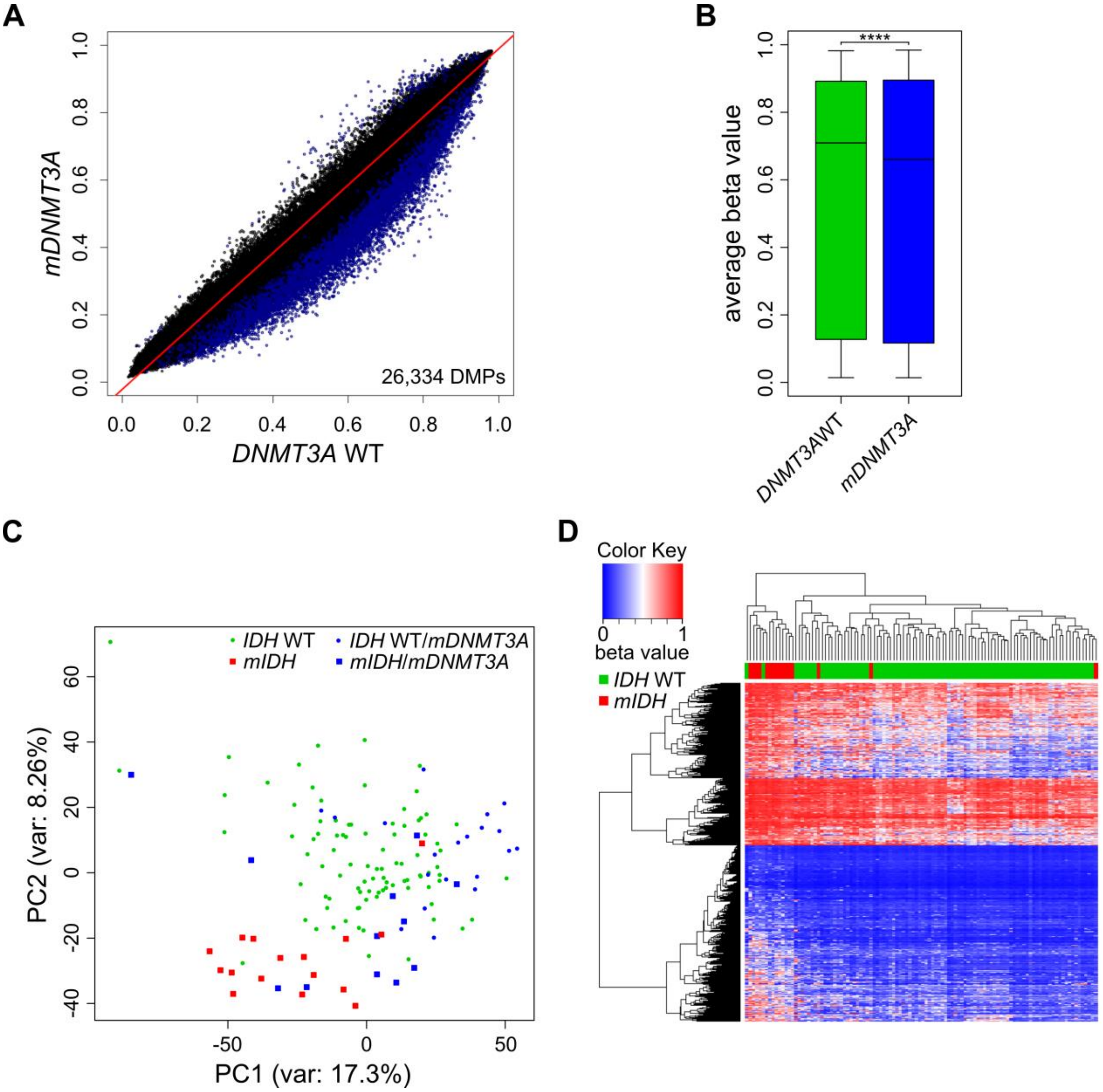

FIGURE S1: Genomic hypermethylation is not a general effect of mutations in epigenetic modifiers in AML. (A) Scatterplot comparing 450K probes of 32 AML patients with mutant *DNMT3A* (*mDNMT3A)* to 108 AML patients with wildtype *DNMT3A (DNMT3A WT)*. Each dot represents one of 421,694 probes left after filtering and blue dots indicate significantly (adj. *P*<0.05) differentially methylated probes (DMPs). **(B)** Average methylation ratios of all analyzed 450K probes in *mDNMT3A* and *DNMT3A* WT AML patients. The difference between the two groups was highly significant (**** *P*<0.0001). **(C)** Principal Component Analysis as in Figure 1D. Patients that had a co-occurring *DNMT3A* mutation are colored in blue. **(D)** Heatmap of 92 *IDH* WT and 16 *mIDH* patients, all of which had *DNMT3A* wildtype status, using the 5000 most significantly differentially methylated probes between the two groups. Each column represents one patient and each row one probe. Dendrograms of patients and probes were obtained using hierarchical clustering by similarity. The color scale indicates beta values.

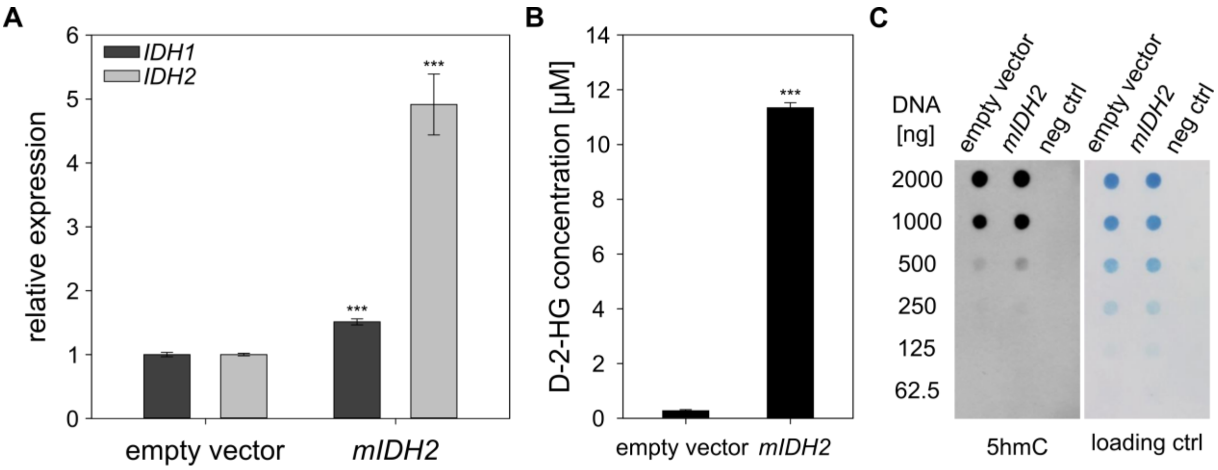

FIGURE S2: Expression of *IDH2* R140Q in cells mimics D-2-HG serum concentrations in *mIDH* AML patients. (A) Expression of *IDH1* and *IDH2* mRNA was measured by qRT-PCR in HL-60 cells transduced with empty vector or *mIDH2* using *ACTB* as a reference gene. Error bars represent standard deviation (n=3). Statistical significance was calculated using Student's *t*-test (*** $P<0.001$). **(B)** D-2-hydroxyglutarate concentration in the cell culture supernatant of HL-60 cells transduced with empty vector or *mIDH2* was determined by an enzymatic assay [74]. Error bars represent the standard deviation (n=3). Statistical significance was calculated using a Welch *t*-test (*** $P<0.001$). **(C)** Immunostaining of DNA dotblots for 5hmC in HL-60 cells expressing *mIDH2*. Sample buffer instead of DNA was loaded as negative control (neg ctrl). Methylene blue staining was used to assess equal loading (loading ctrl).

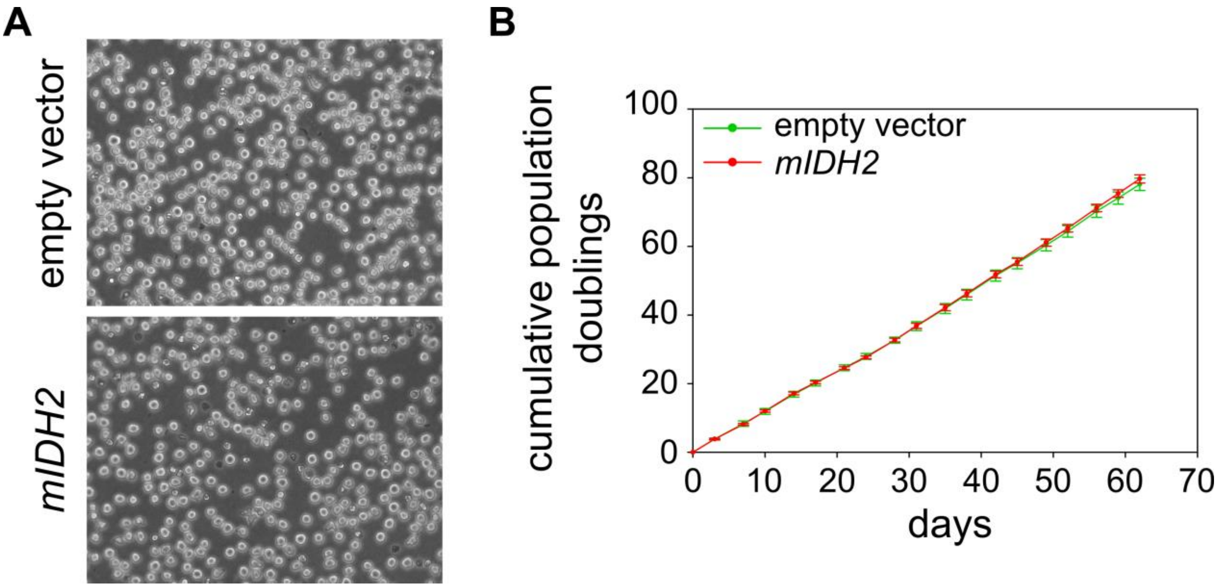

FIGURE S3: Expression of *IDH2* R140Q in HL-60 cells does not lead to overt phenotypic changes. (A) Bright field microscopic images showing the morphology of HL-60 cells transduced with empty vector or *mIDH2*. **(B)** Proliferation analysis of HL-60 cells transduced with empty vector or *mIDH2*. Error bars indicate the cumulative standard deviation after error propagation.

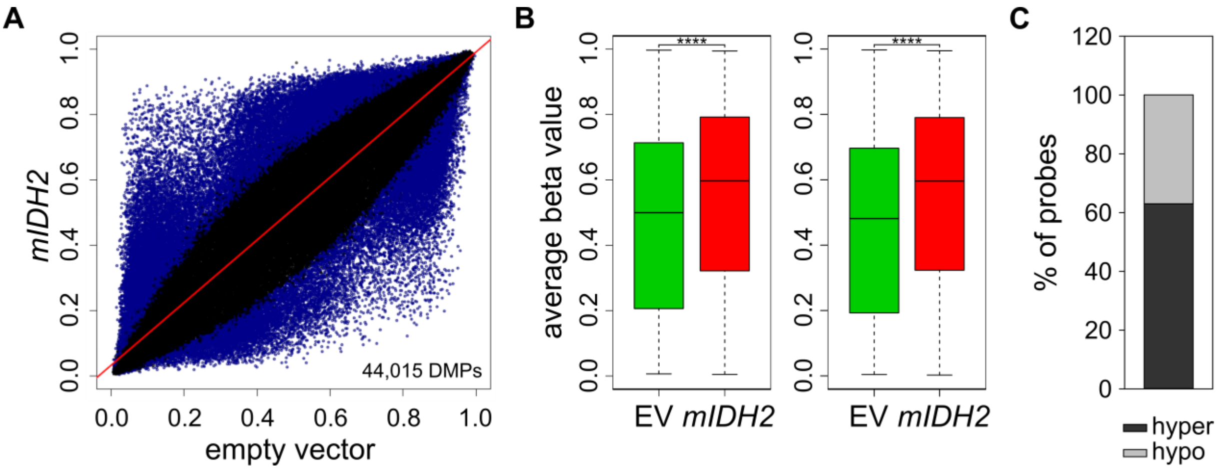

FIGURE S4: Removal of LADs retains hypomethylation in *mIDH2* expressing HL-60 cells. (A) Scatterplots comparing average beta values between HL-60 cells transduced with *mIDH2* or empty vector for all probes common to 450K and EPIC chip with LAD-associated probes being removed. Each dot represents one probe with significantly changed probes (adj. $P<0.05$) colored in blue. **(B)** Boxplots showing average beta values of all significantly differentially methylated probes between *mIDH2* and empty vector (EV) HL-60 cells. All probes common to 450K and EPIC chip without LAD-associated probes were used for the analysis in the left panel. In the right panel all significantly differentially methylated probes irrespective of LAD-association are shown. The difference between the two groups was highly significant (**** $P<0.0001$). **(C)** Barplot showing the percentages of non-LAD hyper- and hypomethylated probes identified in *mIDH2* expressing HL-60 cells.

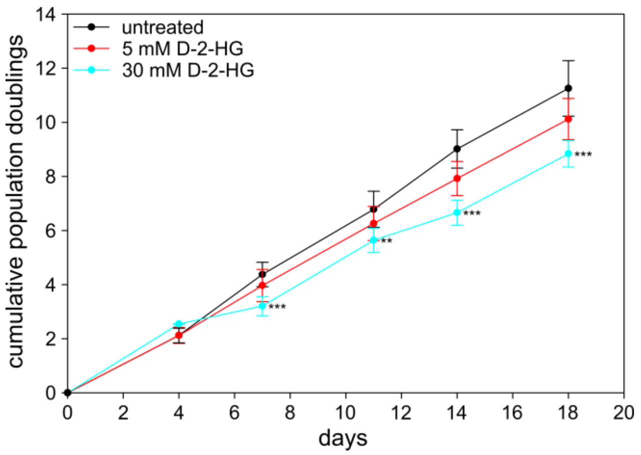

FIGURE S5: Growth curve of D-2-hydroxyglutarate treated HL-60 cells. Proliferation analysis of HL-60 cells treated with different doses of D-2-hydroxyglutarate. Error bars indicate the cumulative standard deviation after error propagation and asterisks show significant changes between untreated and cells treated with 30 mM D-2-hydroxyglutarate (Student's t-test, *** *P*<0.001, ** *P*<0.01)
